# The association between aggregate index of systemic inflammation and DXA-measured body composition parameters in adolescents

**DOI:** 10.3389/fphys.2025.1612735

**Published:** 2025-06-09

**Authors:** Haihua Wang, Luping Tao, Zhongxin Zhu

**Affiliations:** ^1^ Department of Hospital Management, The First People’s Hospital of Xiaoshan District, Xiaoshan Affiliated Hospital of Wenzhou Medical University, Hangzhou, Zhejiang, China; ^2^ Department of Clinical Research Center, The First People’s Hospital of Xiaoshan District, Xiaoshan Affiliated Hospital of Wenzhou Medical University, Hangzhou, Zhejiang, China

**Keywords:** systemic inflammation, adolescents, body composition, muscle mass, visceral adiposity, bone density

## Abstract

**Background:**

Systemic inflammation during adolescence may critically influence metabolic and musculoskeletal health, yet comprehensive biomarkers predicting adverse body composition remain underexplored. The aggregate index of systemic inflammation (AISI), integrating neutrophils, platelets, monocytes, and lymphocytes, offers a novel metric to assess this relationship.

**Methods:**

This cross-sectional study analyzed 3,661 adolescents (aged 12–19 years) from NHANES 2011–2018. AISI was calculated from complete blood counts, and body composition parameters—appendicular lean mass index (ALMI), visceral adipose tissue area (VATA), and total bone mineral density (BMD)—were measured via dual-energy X-ray absorptiometry (DXA). Multivariable linear regression and threshold effect models evaluated associations, adjusting for demographic, metabolic, and lifestyle covariates.

**Results:**

Higher logAISI was associated with lower ALMI (β = −0.189, 95% CI: −0.262 to −0.116), greater VATA (β = 3.017, 1.266–4.769), and reduced BMD (β = −0.017, −0.027 to −0.007). A threshold effect emerged at logAISI = 2.2, beyond which inflammation’s impact on VATA and BMD intensified.

**Conclusion:**

Elevated AISI correlates with adverse body composition in adolescents. The identified threshold suggests a potential clinical benchmark for early intervention. These findings underscore systemic inflammation as a modifiable target to mitigate metabolic and musculoskeletal risks during this critical developmental period.

## Introduction

Adolescence constitutes a critical developmental window characterized by profound physiological transformations that exert enduring effects on metabolic and musculoskeletal health trajectories (Faienza et al., 2023; Wentzel et al., 2025). Emerging evidence reveals an alarming paradigm shift: patterns of muscle deterioration classically associated with senescence now manifest in youth populations, compromising metabolic homeostasis and potentiating insulin resistance (Calcaterra et al., 2024; de Lima et al., 2021). Concurrently, the accumulation of metabolically active adipose tissue precipitates a pro-inflammatory milieu, impairing endocrine signaling and vascular function while amplifying systemic inflammatory markers and early metabolic dysfunction (Dragoumani et al., 2023; Marketou et al., 2023). Equally pivotal is skeletal development during this phase: suboptimal bone mass accrual not only heightens immediate fracture susceptibility but also predisposes individuals to osteoporosis and its sequelae in adulthood (Madhuchani et al., 2023; Sopher et al., 2015). These interdependent perturbations in myocellular integrity, adipocyte distribution, and osteogenic capacity collectively engender a multisystem risk profile, predisposing adolescents to compounded metabolic and musculoskeletal morbidity.

While the inflammation-body composition nexus is well-documented, translational progress remains impeded by the absence of a robust inflammatory metric reliably predictive of clinical outcomes (Calvani et al., 2017). The aggregate index of systemic inflammation (AISI), a composite metric integrating neutrophils, platelets, monocytes, and lymphocytes, emerges as a novel composite biomarker integrating cellular mediators of innate and adaptive immunity (Xiu et al., 2023). Comparative studies suggest AISI’s superior predictive capacity for adverse outcomes across diverse pathologies, attributable to its comprehensive reflection of immune cell interplay (Fois et al., 2020; Ghobadi et al., 2022; Zinellu et al., 2021).

Despite advances in understanding systemic inflammation, critical gaps persist in adolescents. Current research predominantly relies on isolated biomarkers (e.g., CRP, IL-6) (Del Giudice and Gangestad, 2018), which overlook the synergistic interplay of immune cell lineages driving inflammation. While adult studies link inflammation to muscle atrophy (Cheng et al., 2025), visceral adiposity (Varghese et al., 2021), or low bone density (Fuggle et al., 2018), the adolescent context—marked by dynamic tissue plasticity and heightened developmental vulnerability—remains underexplored. This gap limits early interventions during a critical window when body composition trajectories are established.

To address this, we selected dual-energy X-ray absorptiometry (DXA)-derived metrics with mechanistic and clinical relevance: (1) appendicular lean mass index (ALMI), calculated as lean mass in arms and legs/height^2^, aligns with International Society for Clinical Densitometry guidelines (Petak et al., 2013) and is a validated sarcopenia marker (Trimarco et al., 2022), specifically reflecting limb muscle reserves sensitive to inflammatory pathways; (2) visceral adipose tissue area (VATA), a measure of intra-abdominal fat accumulation, was selected for analysis due to its well-established association with systemic inflammation and metabolic dysfunction (Millar et al., 2024; Klein et al., 2025). By integrating ALMI and VATA with total bone mineral density (BMD), this study investigates the association between AISI and body composition parameters during this developmental period.

## Methods

### Study design and population

This cross-sectional study analyzed data from four consecutive cycles (2011–2018) of the National Health and Nutrition Examination Survey (NHANES), a nationally representative program designed to evaluate the health and nutritional status of the U.S. civilian population. NHANES employs a rigorous protocol combining in-depth household interviews, standardized physical examinations in Mobile Examination Centers, and extensive laboratory analyses. All data collection procedures are thoroughly documented, and de-identified datasets are publicly available through the official website (https://wwwn.cdc.gov/nchs/nhanes/), including detailed examination protocols, quality control reports, and analytic guidelines. The study protocol received ethical approval from the National Center for Health Statistics review board, with written informed consent obtained from parents of participants aged 2–17 years, assent from youths aged 7–17 years, and direct consent from those aged 18–19 years (Cheng et al., 2023).

**FIGURE 1:**
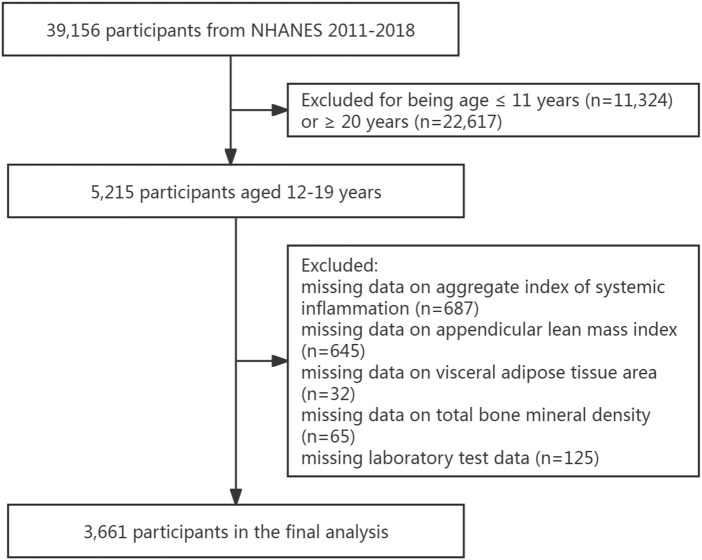
The flowchart of the participants selection.

Our analysis focused on adolescents aged 12–19 years. Following exclusion of individuals with incomplete data for AISI, ALMI, VATA, total BMD, or essential laboratory measures, our final analytical sample included 3,661 participants (Figure 1).

### Study variables

Complete blood counts were quantified using the Beckman Coulter DxH 800 analyzer (Beckman Coulter, Inc.). AISI was computed as (neutrophils × platelets × monocytes)/lymphocytes. Due to right-skewed distributions, AISI values were log-transformed (logAISI) for analyses.

Body composition parameters were assessed using a Hologic QDR 4500A fan-beam densitometer (Hologic, Inc.) by certified technologists in accordance with standardized manufacturer protocols. Data analysis was performed using Hologic Discovery software (version 12.1). Primary outcome measures comprised ALMI, VATA, and total BMD.

Based on established evidence from prior research and clinical considerations, we accounted for potential confounders including: (1) demographic factors–age (continuous), sex (boy/girl), and race/ethnicity (categorized as non-Hispanic White, non-Hispanic Black, Mexican American, or other); (2) socioeconomic status–poverty-income ratio (categorized as low [<1.3], medium [1.3-3.5], or high [>3.5]); (3) anthropometric measure–body mass index (BMI; continuous); (4) lifestyle factors–sedentary behavior (defined as absence of both moderate and vigorous physical activity); laboratory measures (all continuous)–blood urea nitrogen, serum uric acid, serum glucose, total protein, total cholesterol, serum calcium, and serum vitamin D.

### Statistical analyses

Continuous variables were expressed as mean ± standard deviation, while categorical variables were presented as percentages. Group comparisons used ANOVA (normal), Kruskal-Wallis (non-normal), or χ^2^ tests (categorical).

Multivariable linear regression models assessed logAISI-body composition relationships with progressive adjustments: Model 1: adjusted for age, sex, and race; Model 2: additionally adjusted for BMI; Model 3: full adjustment for all covariates. Non-linear relationships were evaluated using generalized additive models with smooth curve fitting. Threshold effects were identified via segmented regression. Subgroup analyses examined effect modification by age, gender, and race/ethnicity.

All analyses were conducted in R v3.4.3 (R Foundation) and EmpowerStats (X&Y Solutions), with statistical significance set at *P* < 0.05 (two-tailed).

## Results

### Baseline characteristics by AISI quartiles

Table 1 presents participant characteristics by AISI quartiles. Age progressively increased across quartiles (15.2–15.7 years), with higher AISI associated with greater proportions of girls (36.7% in Q1 to 55.6% in Q4) and non-Hispanic Black participants (36.4% in Q1 to 14.0% in Q4). Higher quartiles correlated with elevated BMI (22.1–26.2 kg/m^2^), VATA (40.3–58.2 cm^2^) and ALMI (7.18–7.39 kg/m^2^). No significant differences were observed for BMD.

**TABLE 1:** Characteristics of participants based on aggregate index of systemic inflammation quartile.

	Q1	Q2	Q3	Q4	P value
Age (years)	15.2 ± 2.3	15.3 ± 2.3	15.5 ± 2.2	15.7 ± 2.2	<0.001
Sex (%)					<0.001
Boys	63.3	56.6	46.6	44.4	
Girls	36.7	43.4	53.4	55.6	
Race/ethnicity (%)					<0.001
Non-Hispanic White	21.0	28.5	30.8	28.1	
Non-Hispanic Black	36.4	23.1	16.6	14.0	
Mexican American	16.7	21.5	23.3	26.0	
Other race/ethnicity	25.9	26.9	29.3	32.0	
Poverty income ratio (%)					0.022
Low	34.2	40.2	42.0	39.3	
Medium	35.4	34.1	29.9	34.9	
High	20.5	18.1	18.8	16.8	
Unrecorded	9.8	7.5	9.3	9.0	
Sedentary behavior (%)					0.014
Yes	18.0	20.7	20.9	23.7	
No	67.1	63.6	64.8	58.7	
Not recorded	14.9	15.7	14.3	17.6	
Body mass index (kg/m^2^)	22.1 ± 4.8	23.6 ± 5.3	24.6 ± 5.9	26.2 ± 7.1	<0.001
Blood urea nitrogen (mmol/L)	3.9 ± 1.2	4.0 ± 1.3	3.9 ± 1.2	3.9 ± 1.2	0.114
Serum uric acid (umol/L)	292.5 ± 70.5	302.2 ± 73.0	298.4 ± 73.2	306.0 ± 78	<0.001
Serum glucose (mmol/L)	4.9 ± 0.5	4.9 ± 0.6	4.9 ± 0.8	5.0 ± 0.8	0.330
Total protein (g/L)	72.4 ± 4.1	72.4 ± 4.2	72.8 ± 4.2	73.3 ± 4.0	<0.001
Total cholesterol (mmol/L)	4.0 ± 0.8	4.0 ± 0.7	4.0 ± 0.8	4.0 ± 0.8	0.416
Serum calcium (mmol/L)	2.4 ± 0.1	2.4 ± 0.1	2.4 ± 0.1	2.4 ± 0.1	0.061
Serum vitamin D (nmol/L)	55.4 ± 20.6	57.6 ± 20.3	56.6 ± 20.7	56.1 ± 20.5	0.122
Appendicular lean mass index (kg/m^2^)	7.18 ± 1.46	7.23 ± 1.52	7.26 ± 1.57	7.39 ± 1.75	0.028
Visceral adipose tissue area (cm^2^)	40.3 ± 18.6	46.9 ± 23.4	51.2 ± 28.5	58.2 ± 32.8	<0.001
Total bone mineral density (g/cm^2^)	1.04 ± 0.13	1.03 ± 0.12	1.04 ± 0.12	1.04 ± 0.11	0.844

### Multivariable associations between logAISI and body composition

**FIGURE 2:**
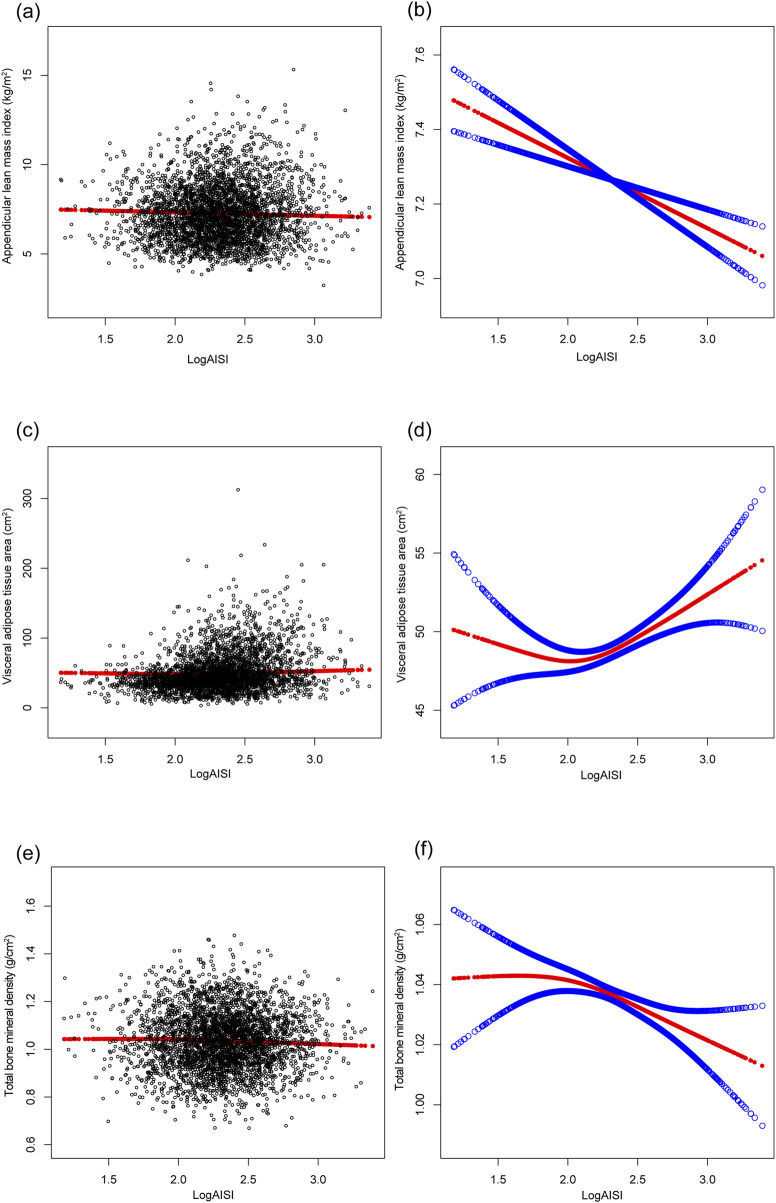
The associations of logAISI with appendicular lean mass index **(a,b)**, visceral adipose tissue area **(c,d)**, and total bone mineral density **(e,f)**. **(a,c,e)** Each black point represents a sample. **(b,d,f)** The solid red line represents the smooth curve fit between variables, and the blue bands denote the 95% confidence interval of the fit. Age, sex, race, poverty income ratio, body mass index, sedentary behavior, blood urea nitrogen, serum uric acid, serum glucose, total protein, total cholesterol, serum calcium, and serum vitamin D were adjusted.

The multivariable analysis (Table 2) revealed dynamic associations: logAISI initially exhibited positive associations with ALMI (β = 0.764, 95%CI: 0.624–0.904) and VATA (β = 20.871, 18.072–23.669), but not BMD (β = 0.009, −0.002–0.019) in Model 1; however, after BMI adjustment (Model 2), these associations reversed for ALMI (β = −0.191, 95%CI: 0.267 to −0.115) and BMD (β = −0.016, −0.027 to −0.006), while the VATA association was attenuated (β = 3.189, 1.412–4.966). In the fully adjusted Model 3, the negative associations persisted for ALMI (β = −0.189, 95% CI: 0.262 to −0.116) and BMD (β = −0.017, 95% CI: 0.027 to −0.007), whereas VATA remained positively associated (β = 3.017, 95% CI: 1.266–4.769). Dose-dependent trends were observed across logAISI quartiles for all three parameters. These associations were confirmed by Figure 2.

**TABLE 2:** Associations of logAISI with appendicular lean mass index, visceral adipose tissue area, and total bone mineral density.

	Model 1 β (95% CI)	Model 2 β (95% CI)	Model 3 β (95% CI)
Appendicular lean mass index	0.764 (0.624, 0.904)***	−0.191 (−0.267, −0.115)***	−0.189 (−0.262, −0.116)***
logAISI			
Q1	Reference	Reference	Reference
Q2	0.280 (0.161, 0.399)	−0.042 (−0.105, 0.021)	−0.051 (−0.111, 0.009)
Q3	0.475 (0.354, 0.595)	−0.034 (−0.099, 0.030)	−0.040 (−0.102, 0.021)
Q4	0.635 (0.514, 0.757)	−0.158 (−0.224, −0.092)	−0.156 (−0.219, −0.093)
P for trend	0.004	<0.001	<0.001
Visceral adipose tissue area			
logAISI	20.871 (18.072, 23.669)***	3.189 (1.412, 4.966)***	3.017 (1.266, 4.769)***
Q1	Reference	Reference	Reference
Q2	6.035 (3.658, 8.411)	0.057 (−1.409, 1.523)	0.117 (−1.322, 1.555)
Q3	10.442 (8.030, 12.854)	0.986 (−0.514, 2.485)	0.952 (−0.521, 2.425)
Q4	17.098 (14.670, 19.527)	2.368 (0.832, 3.905)	2.243 (0.730, 3.756)
P for trend	<0.001	0.001	0.002
Total bone mineral density			
logAISI	0.009 (−0.002, 0.019)	−0.016 (−0.027, −0.006)**	−0.017 (−0.027, −0.007)**
Q1	Reference	Reference	Reference
Q2	0.006 (−0.003, 0.015)	−0.002 (−0.011, 0.007)	−0.003 (−0.011, 0.006)
Q3	0.011 (0.002, 0.020)	−0.002 (−0.011, 0.007)	−0.003 (−0.012, 0.006)
Q4	0.005 (−0.004, 0.014)	−0.016 (−0.025, −0.007)	−0.017 (−0.025, −0.008)
P for trend	0.799	0.001	<0.001

Model 1: age, sex, and race were adjusted.

Model 2: age, sex, race, and body mass index were adjusted.

Model 3: age, sex, race, poverty income ratio, body mass index, sedentary behavior, blood urea nitrogen, serum uric acid, serum glucose, total protein, total cholesterol, serum calcium, and serum vitamin D were adjusted.

*P < 0.05, **P < 0.01, ***P < 0.001.

AISI: aggregate index of systemic inflammation.

### Stratified analyses by demographic subgroups

**FIGURE 3:**
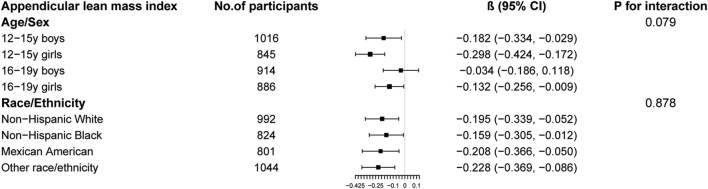
Subgroup analysis of the association between logAISI and appendicular lean mass index. Age, sex, race, poverty income ratio, body mass index, sedentary behavior, blood urea nitrogen, serum uric acid, serum glucose, total protein, total cholesterol, serum calcium, and serum vitamin D were adjusted. In the subgroup analysis, the model is not adjusted for the stratification variable itself.

**FIGURE 4:**
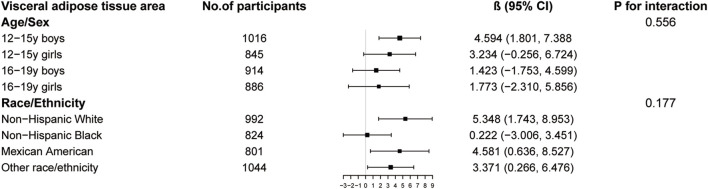
Subgroup analysis of the association between logAISI and visceral adipose tissue area. Age, sex, race, poverty income ratio, body mass index, sedentary behavior, blood urea nitrogen, serum uric acid, serum glucose, total protein, total cholesterol, serum calcium, and serum vitamin D were adjusted. In the subgroup analysis, the model is not adjusted for the stratification variable itself.

**FIGURE 5:**
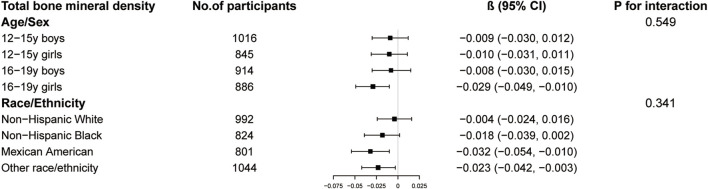
Subgroup analysis of the association between logAISI and total bone mineral density. Age, sex, race, poverty income ratio, body mass index, sedentary behavior, blood urea nitrogen, serum uric acid, serum glucose, total protein, total cholesterol, serum calcium, and serum vitamin D were adjusted. In the subgroup analysis, the model is not adjusted for the stratification variable itself.

The results of subgroup analysis are shown in Figures 3–5. For ALMI (Figure 3), inverse associations were consistent across all subgroups, with the strongest effect observed in younger girls (β = −0.298, 95% CI: 0.424 to −0.172). VATA (Figure 4) showed the most pronounced positive associations in younger boys (β = 4.594, 95% CI: 1.801–7.388) and non-Hispanic Whites (β = 5.348, 95% CI: 1.743–8.953). Total BMD (Figure 5) exhibited significant negative associations, particularly among older girls (β = −0.029, 95% CI: 0.049 to −0.010) and Mexican Americans (β = −0.032, 95% CI: 0.054 to −0.010).

### Threshold effects

Threshold analysis revealed a significant inflection point at logAISI = 2.2 for both VATA and BMD (Table 3). Below this threshold, logAISI showed no significant association with VATA (β = −2.189, 95% CI: −6.089 to 1.711) or BMD (β = 0.002, 95% CI: −0.020–0.025). Above 2.2, logAISI positively correlated with VATA (β = 5.982, 95% CI: 3.336–8.628) and inversely with BMD (β = −0.028, 95% CI: −0.044 to −0.013).

**TABLE 3:** Threshold effect analysis of logAISI on visceral adipose tissue area and total bone mineral density.

logAISI	Adjusted ß (95% CI), p-value
Visceral adipose tissue area	
Inflection point	2.2
logAISI <2.2	−2.189 (−6.089, 1.711), 0.271
logAISI >2.2	5.982 (3.336, 8.628), <0.001
Log likelihood ratio	0.003
Total bone mineral density	
Inflection point	2.2
logAISI <2.2	0.002 (−0.020, 0.025), 0.840
logAISI >2.2	−0.028 (−0.044, −0.013), <0.001
Log likelihood ratio	0.060

Age, sex, race, poverty income ratio, body mass index, sedentary behavior, blood urea nitrogen, serum uric acid, serum glucose, total protein, total cholesterol, serum calcium, and serum vitamin D were adjusted.

## Discussion

Our findings demonstrate that higher AISI is robustly associated with adverse body composition in adolescents, with lower ALMI, greater VATA, and reduced BMD. Notably, we identified a critical inflection point (logAISI = 2.2), beyond which inflammatory burden exerted exponentially greater effects on VATA and BMD. These findings suggest that systemic inflammation may contribute to concurrent metabolic and musculoskeletal dysregulation during adolescence.

Systemic inflammation is a hallmark of sarcopenia, with elevated inflammatory markers linked to muscle mass loss and functional decline in adults (Zhu et al., 2024; Byun et al., 2017). Pro-inflammatory cytokines, such as those activating NF-κB signaling, disrupt the equilibrium between muscle protein synthesis and degradation, exacerbating sarcopenia progression (Webster et al., 2020; Li et al., 2008). Concurrently, chronic inflammation suppresses anabolic hormones like growth hormone and insulin-like growth factor-1, impairing myocyte maintenance and protein synthesis (Mellen et al., 2023). Our results extend this paradigm to adolescents, suggesting that inflammatory muscle loss may initiate decades earlier than traditionally assumed, potentially compromising peak muscle reserve critical for metabolic health in adulthood.

Adipose tissue functions as both a source and a consequence of systemic inflammation. Its expansion drives chronic low-grade inflammation characterized by infiltrating pro-inflammatory immune cells (e.g., macrophages, T lymphocytes) and elevated cytokines (e.g., TNF-α, IL-6, CRP) (Kredel and Siegmund, 2014; Kawai et al., 2021). This milieu disrupts adipocyte function, promoting lipolysis, oxidative stress, and a self-perpetuating cycle of inflammation (Kolb, 2022; Šebeková et al., 2023). Notably, our study identified a threshold effect, beyond which inflammation disproportionately exacerbates visceral adiposity. This threshold may represent a critical point for clinical intervention, particularly given the concomitant suppression of anti-inflammatory adipokines like adiponectin, which exacerbates insulin resistance and further adipose accumulation (Berg and Scherer, 2005; Ghigliotti et al., 2014).

The inverse association between AISI and BMD aligns with evidence that systemic inflammation disrupts bone remodeling (Terkawi et al., 2022; Epsley et al., 2020). Chronic inflammation skews the osteoclast-osteoblast balance, favoring excessive resorption while impairing formation, ultimately reducing bone density (Singh et al., 2023; Clowes et al., 2005). A prospective cohort study in adolescent girls demonstrated a significant inverse correlation between subclinical inflammation and BMD, with overweight individuals showing particularly marked effects (Lucas et al., 2012). The pathophysiology involves adipokine dysregulation, wherein pro-inflammatory cytokines promote osteoclastogenesis while suppressing osteoblast activity, tilting the bone remodeling balance toward net resorption (Lee et al., 2023). These mechanistic insights highlight the potential of anti-inflammatory strategies—such as dietary adjustments, pharmacological interventions, and physical activity—as viable therapeutic options for preserving bone health in adolescents with elevated inflammation levels (Rizzoli and Chevalley, 2024; Schett, 2011; Jones et al., 2014).

To our knowledge, this is the first study to delineate AISI’s associations with body composition in adolescents, bridging gaps between adult sarcopenia, obesity, and osteoporosis research. AISI offers a cost-effective tool to identify at-risk adolescents early, with the inflection point serving as a potential clinical benchmark. However,several limitations should be noted. First, its observational design precludes causal inference between AISI and body composition parameters; longitudinal or interventional studies are required to establish temporality and mechanistic pathways. Second, despite extensive covariate adjustment, residual confounding from unmeasured factors (e.g., genetic predispositions, early-life environmental exposures, or pubertal hormone levels) may persist. Third, while AISI is integrative, it does not account for non-cellular inflammatory mediators or tissue-specific inflammation, which could offer additional pathophysiological insights. Fourth, while we adjusted for BMI to account for potential confounding, its role as a mediator in the causal pathway requires consideration. This adjustment may have: (1) partially obscured the true effect of inflammation on visceral adiposity through overadjustment, and (2) artificially reversed the direction of associations with lean mass and bone density. The complex interplay between BMI and inflammation underscores the necessity of longitudinal designs with formal mediation analyses to elucidate these temporal and mechanistic relationships.

## Conclusion

This study reveals significant associations between AISI and adverse body composition parameters in adolescents, characterized by reduced lean mass, elevated visceral adiposity, and lower bone density. The identified threshold (logAISI = 2.2) suggests a clinically relevant inflammatory benchmark that may help stratify adolescents at risk for concurrent metabolic and musculoskeletal dysregulation. Future research should validate these associations in prospective cohorts and explore whether interventions targeting inflammation can modify body composition trajectories in this population.

## Data Availability

The datasets presented in this study can be found in online repositories. The names of the repository/repositories and accession number(s) can be found below: The data of this study are publicly available on the NHANES website (https://wwwn.cdc.gov/nchs/nhanes/Default.aspx).
